# The Impact of Evaluation Strategy on Sepsis Prediction Model Performance Metrics in Intensive Care Data: Retrospective Cohort Study

**DOI:** 10.2196/72083

**Published:** 2026-03-24

**Authors:** Dang-Khoa Do, Patrick Rockenschaub, Sebastian Daniel Boie, Oliver Kumpf, Hans-Dieter Volk, Felix Balzer, Falk von Dincklage, Gregor Lichtner

**Affiliations:** 1 Institute of Medical Informatics Charité – Universitätsmedizin Berlin, Corporate Member of Freie Universität Berlin and Humboldt-Universität zu Berlin Berlin Germany; 2 Institute of Clinical Epidemiology, Public Health, Health Economics, Medical Statistics and Informatics Medical University of Innsbruck Innsbruck Austria; 3 Department of Anesthesiology and Intensive Care Medicine (CCM/CVK) Charité – Universitätsmedizin Berlin, Corporate Member of Freie Universität Berlin and Humboldt-Universität zu Berlin Berlin Germany; 4 Institute of Medical Immunology Charité – Universitätsmedizin Berlin, Corporate Member of Freie Universität Berlin and Humboldt-Universität zu Berlin Berlin Germany; 5 Department of Anesthesia, Critical Care, Emergency and Pain Medicine Universitätsmedizin Greifswald Greifswald Germany

**Keywords:** sepsis, machine learning, deep learning, intensive care, intensive care unit, ICU, early prediction, early warning, electronic health records, prediction, generalizability

## Abstract

**Background:**

The prediction of the onset of sepsis, a life-threatening condition resulting from a dysregulated response to an infection, is one of the most common prediction tasks in intensive care–related machine learning research. To assess the performance of such models, different evaluation strategies, including fixed horizon (a single prediction at a set time before onset), peak score (a single prediction using the maximum predicted risk across time), and continuous evaluation (multiple predictions assessed continuously across time), are commonly implemented, but there is no clear consensus on which approach should be used in order to provide clinically meaningful performance evaluation.

**Objective:**

This study aimed to assess different evaluation approaches of sepsis prediction models trained on a public intensive care dataset applied to German intensive care data.

**Methods:**

In this retrospective, observational cohort study, we assessed the efficacy of machine learning models, pretrained on the Medical Information Mart for Intensive Care IV dataset, when applied to BerlinICU, a multisite German intensive care dataset. To understand the real-world impact of implementing these models, we examined the performance variability across various evaluation strategies.

**Results:**

The BerlinICU dataset includes 40,132 intensive care admissions spanning 10 years (2012-2021). Using the latest Sepsis-3 definition, we identified 4134 septic admissions (10.3% prevalence). Application of a temporal convolutional network model to BerlinICU yielded an area under the receiver operating characteristic curve (AUROC) of 0.67 (95% CI 0.66-0.68) for continuous evaluation with a 6-hour prediction horizon, compared with 0.84 (95% CI 0.83-0.85) on the test set of Medical Information Mart for Intensive Care IV. On BerlinICU, peak score evaluation showed a similar AUROC compared with continuous evaluation, while fixed horizon evaluation showed a reduced AUROC of 0.61 (95% CI 0.60-0.62). Onset matching had minimal impact on performance estimates using continuous evaluation or fixed horizon evaluation, but increased estimates for peak score evaluation. Performance metrics improved with shorter prediction horizons across all strategies.

**Conclusions:**

Our results demonstrate that the choice of evaluation strategy has a significant impact on the performance metrics of intensive care prediction models. The same model applied to the same dataset yields markedly different performance metrics depending on the evaluation approach. Therefore, careful selection of the evaluation approach is essential to ensure that the interpretation of performance metrics aligns with clinical intentions and enables meaningful comparisons between studies. In our view, the continuous evaluation approach best reflects the continual monitoring of patients that is performed in real-world clinical practice. In contrast, fixed-horizon and peak score evaluation approaches may produce skewed results when not properly matching the length of stay distributions between sepsis cases and control cases. Especially for peak score evaluation, longer visits tend to produce higher maximum scores because sampling from more values increases the likelihood of capturing higher values purely by chance.

## Introduction

Sepsis is a life-threatening condition resulting from a dysregulated response to an infection that leads to organ dysfunction [1], responsible for up to 20% of all deaths worldwide [2]. Beyond its significant mortality rate [3], sepsis represents a tremendous financial burden, totaling to almost US $24 billion in the United States in 2013 [4]. Those who survive may experience long-term health consequences, including a diminished quality of life [5].

As each hour of delayed therapy increases mortality [6-8], sepsis treatment guidelines continue to recommend early therapy within the first hour of sepsis recognition [9-11]. Therefore, an early recognition of sepsis is essential. Numerous studies have investigated machine learning to aid early prediction of sepsis in the intensive care unit (ICU) [12,13], including prospective application [14,15] and external validation on data of different hospitals [14,16-18]. However, the strategies used in previous studies to evaluate model performance vary widely, particularly in the criteria for data selection and how predictions are aligned with respect to the sepsis onset. The most commonly used evaluation strategies can be broadly categorized into three groups, (1) fixed horizon evaluation, which assesses the model’s ability to predict sepsis at a fixed number of hours before onset [13,19]; (2) peak score evaluation, which uses the highest prediction score across time before onset [16,20]; and (3) continuous evaluation, which considers all prediction scores across time before onset [13]*.*

While these strategies are widely used in the literature, it remains uncertain how the choice among them influences the estimated model performance and whether certain strategies may systematically inflate or deflate metrics compared with others, even when applied to the exact same model and dataset. This has direct implications for clinical deployment and benchmarking: if a model is selected based on an evaluation strategy that does not reflect its intended clinical use (eg, hourly real-time monitoring vs one-time assessment), its actual performance may fall short of expectations. Similarly, comparing models across studies without accounting for differences in evaluation strategy risks drawing misleading conclusions about their relative performance.

In addition to differences in evaluation strategy, the literature also uses a range of performance metrics. Standard measures include the area under the receiver operating characteristic curve (AUROC), area under the precision-recall curve (AUPRC), and threshold-based metrics, such as positive predictive value (PPV) and negative predictive value (NPV). Threshold-based metrics reflect clinical utility at specific decision points, which is critical for deployment. In contrast, AUROC is threshold-agnostic, capturing model discrimination across all thresholds, and is robust to class imbalance, which makes it the predominant choice in the literature [21]. Beyond standard performance metrics, recent sepsis-prediction challenges have adopted clinically informed cost functions that capture specific clinical trade-offs. The PhysioNet/CinC 2019 challenge used a time-weighted continuous utility score, rewarding early detection while penalizing very early, late, and false alarms across the entire ICU trajectory. In contrast, the Pediatric Sepsis Data Challenge evaluates a single admission-time prediction per patient and scores models by their true-positive rate at a fixed maximal false-positive rate [22,23]. These examples highlight that not only evaluation strategies, but also the choice of performance metric, can fundamentally shape how sepsis prediction models are interpreted and compared.

In this study, we aimed to investigate how the choice of the evaluation approach influences the estimated performance of sepsis prediction models in an external validation context. Specifically, we compared the performance of models trained on a public intensive care dataset from the United States and subsequently applied them to German intensive care data. In doing so, we also address the lack of studies involving large-scale ICU cohorts from the German health care context, where the efficacy of sepsis prediction models—given the distinct patient demographics, treatment approaches, and health care policies—remains largely uninvestigated.

## Methods

### Study Design and Setting

This is a retrospective cohort study for evaluating differences in performance metrics under different, commonly used evaluation strategies. Temporal convolutional network (TCN) models were developed on Medical Information Mart for Intensive Care IV (MIMIC-IV v2.0; USA) and applied to BerlinICU, a cohort of adult ICU stays from 8 ICUs at Charité—Universitätsmedizin Berlin across 2 sites (Campus Virchow Klinikum and Campus Charité Mitte) from 2012 to 2021. No clinical intervention or change in patient management was implemented; all data were collected during routine care.

### Participants and Flow

We included ICU stays from adult patients (≥18 y). We excluded stays originating from non-ICU wards, stays with <6 hours of data, gaps ≥12 hours, sepsis onset within 4 hours after admission, and truncated each stay at 7 days. Evaluation samples for different evaluation strategies were drawn from this cohort.

### Primary and Secondary Outcome Measures

The clinical outcome was sepsis onset, defined according to Sepsis-3 with an adapted suspicion-of-infection definition for BerlinICU (refer to “Outcome definition” section). The primary performance measure used to compare evaluation strategies was the AUROC. Secondary performance measures included the AUPRC, PPV, NPV, and recall at fixed thresholds. We additionally report “lifted” PPV and AUPRC (each divided by the horizon-specific prevalence) and recall at the threshold where precision equals twice the prevalence, as well as Brier score, alarm rate, and net benefit to assess calibration and clinical utility.

### Datasets

We pretrained models on the MIMIC-IV dataset (version 2.0), a well-known ICU dataset from the United States that is commonly used to develop clinical prediction models [12,13]. We applied these models to BerlinICU, a comprehensive dataset from 8 ICUs from Charité - Universitätsmedizin Berlin in Germany, one of Europe’s largest university hospitals. The ICUs were distributed across 2 different sites (Campus Virchow Klinikum and Campus Charité Mitte).

BerlinICU was extracted from the intensive care data management systems and the hospital information system present at the Charité - Universitätsmedizin Berlin. Specifically, we extracted the same clinical features that we used to pretrain the models, resulting in a total of 48 time-varying features, including vital parameters and laboratory results, along with 4 static features (age, sex, height, and weight; Table S1 in Multimedia Appendix 1). These features comprise demographic variables, vital signs, arterial blood gas values, electrolyte and blood count parameters, as well as treatment-related variables (eg, fraction of inspired oxygen) and clinical output measures (eg, urine output). All clinical feature values outside of physiologically plausible ranges, as defined by the ricu package [24], were removed. The remaining data was aggregated into intervals of one hour using their median value. Missing values were imputed using a forward fill strategy. If no previous values were available, the training set mean across all patients, regardless of sepsis status, for that feature was used. This corresponds to zero imputation if performed after feature standardization and is common practice in machine learning studies on sepsis prediction [17,25,26]. Outlier influence was mitigated by previous filtering based on physiological plausibility. Data were excluded from the dataset if they originated from nonintensive care wards, involved patients younger than 18 years, had a sepsis onset within 4 hours after admission, contained less than 6 hours of data, or included gaps equal to or exceeding 12 hours. The dataset was restricted to data from the years 2012-2021 and limited to at most 7 days following admission to the ICU.

### Ethical Considerations

This study was approved by the local ethics committee (Ethikausschuss am Campus Virchow-Klinikum, Charité—Universitätsmedizin Berlin, Chairperson PD Dr E Kaschina, application EA2/137/22, approval date July 12, 2022, and amendment date May 10, 2023). Informed consent was not required due to the retrospective, observational nature of the study and the use of routinely collected data in accordance with applicable regulations. No compensation was provided.

### Outcome Definition

**Figure 1:**
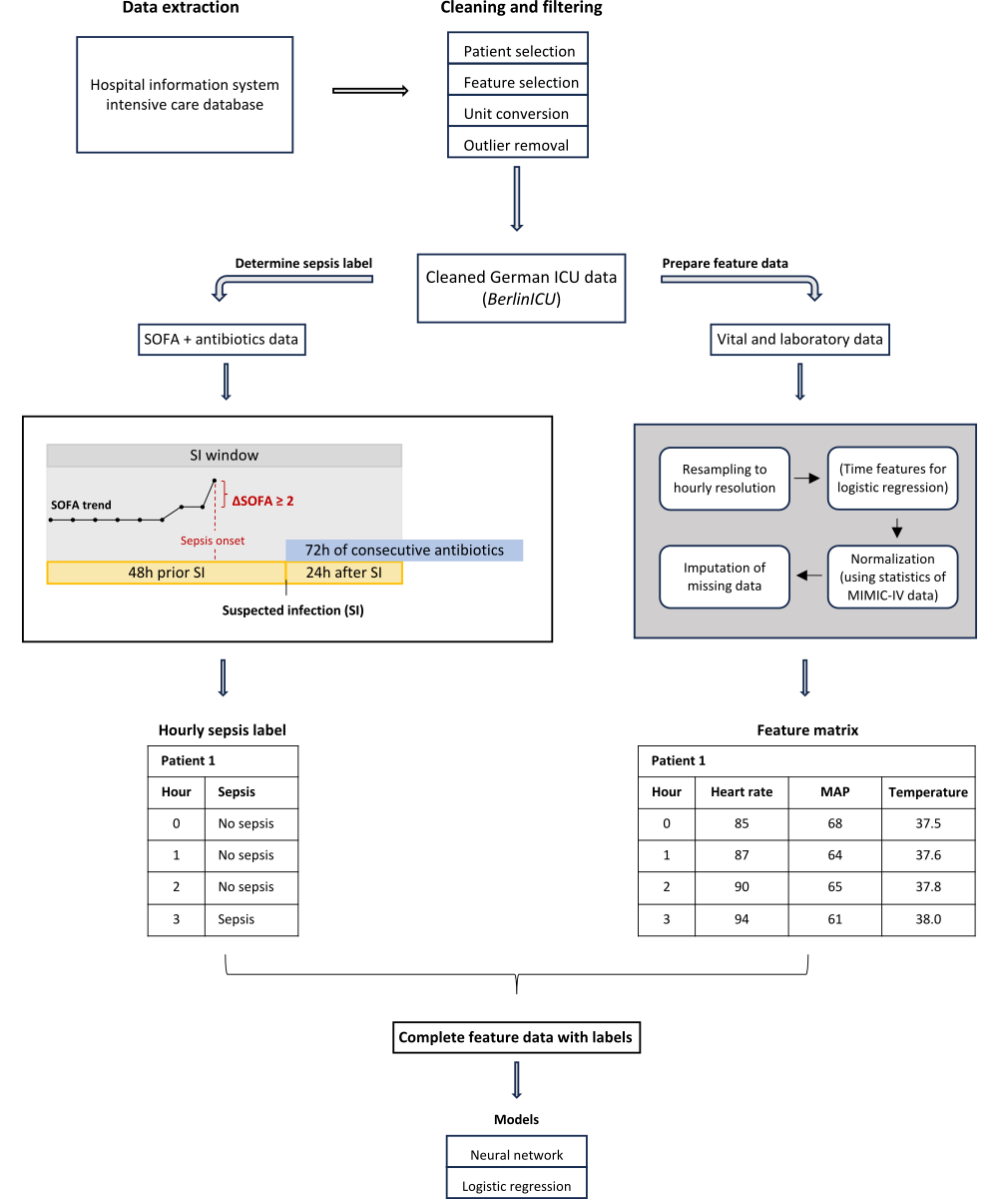
Preprocessing Pipeline. The BerlinICU dataset was extracted from the hospital information system and cleaned. We inferred sepsis onset according to the Sepsis-3 definition. For the logistic regression baseline models, we created additional temporal features. The final dataset was then passed to the models and evaluated. ICU: intensive care unit; MAP: mean arterial pressure; MIMIC-IV: Medical Information Mart for Intensive Care IV; SI: suspicion of infection; SOFA: Sequential Organ Failure Assessment score.

To identify the onset of sepsis, we used the latest Sepsis-3 definition criteria, which require the co-occurrence of a suspicion of infection (SI) and an increase of the Sequential Organ Failure Assessment (SOFA) score by at least 2 points [1]. An SI is present when antibiotic treatment is coupled with microbiological culture tests. As the BerlinICU dataset did not include microbiological data, we used an alternative definition for SI in line with previous studies [16,17]. We inferred SI if a patient was treated with antibiotics administered at least once every 24 hours for at least 3 days, and defined the time of suspicion as the beginning of this treatment. Following previous studies, we defined an SI window ranging from 48 hours before to 24 hours after the time of SI (Figure 1). We assumed the onset of sepsis if an increase of at least 2 points of the SOFA score occurred during that SI window. The sepsis onset time point was defined as the time point of the recorded SOFA increase.

### Model Training

We used TCN models trained on the MIMIC-IV dataset from our previous study [17]. This choice is guided by a comprehensive study by Bai et al [27], which demonstrated the superior performance of the TCNs in sequential data in various tests and domains, and by our own previous work, in which TCNs showed consistently strong or superior performance compared with gated recurrent units (GRUs) and attention-based architectures across multiple public ICU datasets [17]. The TCN architecture uses causal convolutions with stride 1, padding to preserve sequence length, and exponentially increasing dilation to expand the receptive field. Each convolution is followed by a Chomp1d operation to enforce causality, Leaky ReLU activation, dropout, and residual connections. Our models were trained to predict, for each hourly bin, the individual risk of sepsis onset in the next 6 hours. We used a 10-times repeated random split scheme for hyperparameter tuning, resulting in 10 trained models for the hyperparameter set with the lowest average validation loss. For each repetition in this scheme, 20% of the data were held out as a test set. The remaining data were split into training (64%) and validation (16%). For each repetition, 10 randomly drawn hyperparameter configurations were evaluated. The configuration with the lowest average validation loss across the 10 repetitions was selected to train the final models. Training was performed using a binary cross-entropy loss, where class weights were calculated based on the inverse prevalence of the positive class in the training data, an Adam optimizer for a maximum of 1000 epochs, and early stopping with a patience of 10 epochs based on validation loss. The randomized hyperparameter search was conducted on learning rate (sampled log-uniform from exp(–10) to exp(–3)), weight decay (0, 1e–7, 1e–6, …, 1), dropout probability (0.3, 0.4, …, 0.7), batch size (128, 256, and 512), hidden dimension (32, 64, and 128), number of layers (1, …, 10), and kernel size (2, …, 6). A detailed description of the architecture, hyperparameter search, and validation procedure is provided in our previous publication [17]. To investigate a possible dependence of model architecture on the results, we also trained GRU and attention-based models on the MIMIC-IV dataset for comparison. Model training and evaluation followed the same procedures as described in our previous publication [17].

To enhance model performance, binary missingness indicators were added for the 48 time-varying and 4 static features used as inputs. This representation has been shown to capture clinically meaningful information about testing behavior but may also encode site-specific practice patterns [22,28]. To mitigate overfitting, models were trained with regularization (weight decay) and early stopping. The final models, along with their tuned hyperparameters, were then applied to the BerlinICU dataset without any further fine-tuning or retraining.

For establishing baselines in our analyses, we used logistic regression models as implemented in scikit-learn with default settings, corresponding to L2-regularized logistic regression, without hyperparameter tuning. These were trained following a 5-fold cross-validation scheme in which 80% of the data were used for training and 20% for testing. To better capture the temporal dynamics, we additionally engineered temporal features for the 48 time-varying features. These temporal features represent the trajectory of the measurements over time and include the minimum, maximum, mean, median, and variance of the last 4, 8, and 16 hours for the 48 laboratory and vital features, totaling 772 features (48 time-varying features from the hourly bins, their 720 temporal features, and 4 static features). Although this results in a large number of features relative to the number of events, these features are highly correlated temporal summaries, and penalization reduces the effective model complexity. To account for the class imbalance, we used class weights inversely proportional to class frequencies.

Training and evaluation were performed using Python 3.10.8, PyTorch 1.12.1, and scikit-learn 1.1.3.

### Model Evaluation

**Figure 2:**
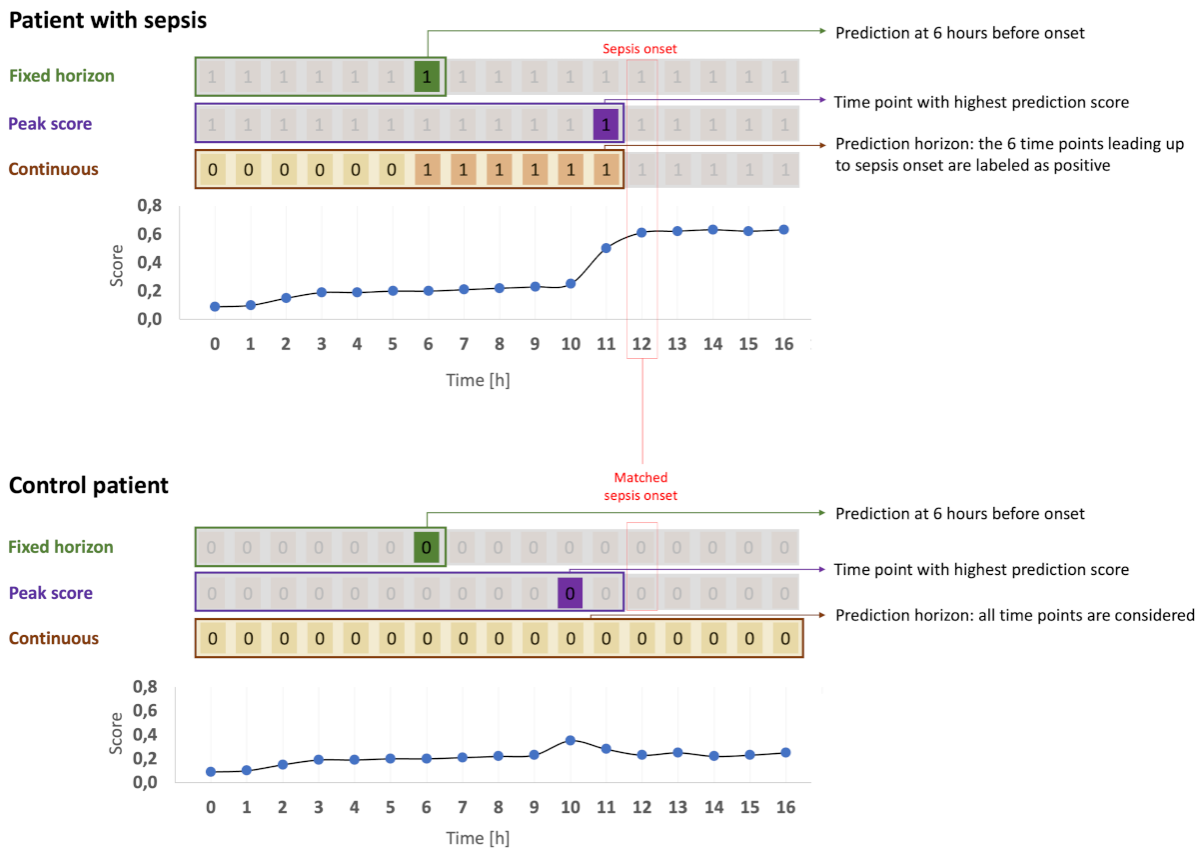
Overview of the evaluation strategies. For the fixed horizon evaluation, a prediction horizon was defined, where predictions were made for a time point that preceded the onset (or control onset), a predefined number of hours (6 hours in this example). Only the data from admission up to this time point was used to make the prediction (green rectangle). Labels were assigned as “positive” (marked as green “1”) for patients with sepsis or “negative” (marked as green “0”) for control patients. In the peak score evaluation (purple rectangle), the maximum prediction score across all time points before onset was determined. Patients with sepsis were labeled “positive,” while control patients were labeled “negative” (purple “1” or “0” label). In the continuous evaluation (brown rectangle), for patients with sepsis, timepoints within a specific time window before the onset (6 hours in this example) were labeled “positive” (brown “1”s), while earlier time points—and all timepoints for control patients—were labeled “negative” (light brown “0”s). All timepoints before the onset (or control onset) were considered for evaluation. For patients with sepsis (top), the onset was the actual sepsis onset. For control patients (bottom), the onset was defined either as a hypothetical control onset matched to the onset time of a patient with sepsis (“matched onset”) or as the last available data point, either at discharge or at 7 days after admission, whichever occurred first. The rectangular frames represent the data that was ultimately considered for each evaluation strategy. Shown here are the default settings for each evaluation strategy: the fixed horizon and peak score evaluations use matched sepsis onset times, while the continuous evaluation is depicted without onset matching. Note that the underlying patient data are the same across all panels; only the labeling and selected time points differ according to the respective evaluation strategy.

To focus on evaluating the real-world applicability of the models in a clinical setting, we implemented 3 different evaluation strategies that scrutinized different aspects of the models’ performance—fixed horizon evaluation, peak score evaluation, and continuous evaluation. Each strategy reflects a different assumption about how a model is applied in practice, and thus, yields performance metrics with different interpretations (Figure 2).

### Onset Matching

A key aspect of the evaluation strategies is the handling of time series data for patients without sepsis (controls). While patients with sepsis (cases) have a clearly defined sepsis onset that marks the end point of their time series for analysis, patients without sepsis lack such a reference point. Therefore, we used 2 commonly used approaches to define the end point of their time series for analysis. First, no onset matching, in which the hypothetical onset for control patients is defined as the last available data point, either at discharge or at 7 days after admission, whichever occurred first. Second, onset matching, in which onsets of control patients are aligned with those of patients with sepsis by randomly pairing each control patient with a patient with sepsis whose time to sepsis onset does not exceed that of the control patient. The time from admission to sepsis onset in the matched patient with sepsis is then used as the hypothetical onset time for the control patient [16,29,30]. This approach preserves alignment to admission for both groups and ensures that the distribution of evaluation time points is comparable between patients with sepsis and patients without sepsis, thereby reducing bias due to differences in lengths of stay.

### Fixed Horizon Evaluation

This approach evaluates a model’s ability to predict the onset of sepsis at a predefined time point before the actual onset—the so-called prediction horizon (*h*)—using all available data up to that time [13,19]. Varying the length of the prediction horizon allows for assessing the earliness of the model’s predictive ability.

The predicted score *y_pred_*, which is based on all available data for the patient up to *h* hours before the sepsis onset for patients with sepsis or up to a hypothetical onset for control patients (*t_onset_*), and the true label *y_true_* are given by:

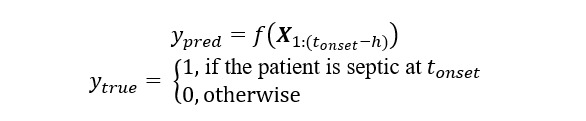



where ***X***_1:_*_t_* is the patient data (feature matrix) from time point 1 to *t*, and *f* is the model that outputs a score correlated to the probability of a sepsis onset.

### Peak Score Evaluation

Peak score evaluation is based on the assumption that a prediction model in a clinical setting operates with a fixed threshold, and once any prediction score surpasses this threshold, an alarm for that particular patient is triggered [16,20]. To capture this behavior, the maximum model prediction score from ICU admission to the onset of sepsis or control offset is determined, resulting in a single score-label pair for each patient for performance score calculation. Here, the prediction score and true label are given by:

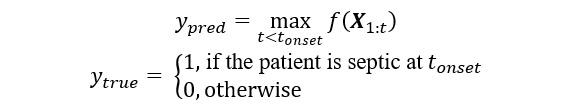



For horizon-dependent analysis, we used the peak score within the defined horizon instead of across all time points of each patient.

### Continuous Evaluation

This approach evaluates a series of predictions made continuously for each patient over time [13]. Specifically, predictions for all time points before sepsis onset (or control onset) are considered. The resulting performance metrics from this approach represent the model’s predictive efficiency across all time points, simulating its use for a randomly selected patient at a random time point.

Using this approach included first establishing a specific time horizon (eg, 6 h) to assess the model’s ability to predict the sepsis onset at any given time point within that timeframe. For patients with sepsis, a positive label was assigned to all time points within the horizon, while all other time points were labeled as negative. For control patients, all time points were labeled as negative. Importantly, the time horizon only determines which time points before onset are labeled as positive, but does not restrict the range of time points included in the evaluation. We included the prediction scores from each hourly interval up to the onset of sepsis in patients with sepsis or the hypothetical control onset in control patients (*t_onset_*), excluding the prediction at the actual onset (or control onset in case of onset matching). The prediction score and true label are given by:

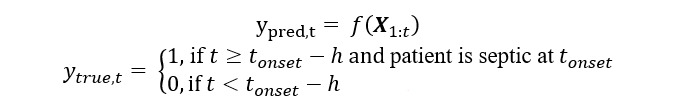



This procedure generated a sequence of hourly score-label pairs for each patient:

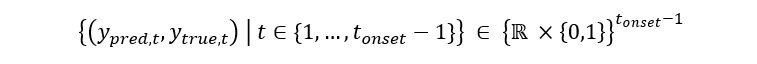



To accommodate variations in the length of these sequences among patients, we applied inverse frequency weighting to the samples when computing performance metrics.

### Performance Metrics

For all strategies, we report the AUROC. We chose AUROC because, unlike other commonly applied metrics such as AUPRC, accuracy, precision or PPV, NPV, or *F* score, the AUROC is independent of sepsis prevalence. This is essential for our study, since sepsis prevalence varies not only between the 2 datasets (MIMIC-IV vs BerlinICU) but also within a dataset across evaluation strategies; for example, in continuous evaluation, the proportion of positive time points changes with each prediction horizon. Using a prevalence-sensitive metric under these conditions would confound discrimination performance with shifts in prevalence and, therefore, blur the effect we wish to study. AUROC instead measures the probability that the model ranks a randomly chosen positive instance higher than a randomly chosen negative one, providing a prevalence-robust estimate of discrimination across all decision thresholds. However, to support interpretability, we additionally report AUPRC, PPV, NPV, and sensitivity at fixed thresholds (Figures S5 and S6 in Multimedia Appendix 1).

Because AUPRC, PPV, and NPV depend on event prevalence, we harmonized the patient-level prevalence across strategies and horizons in all evaluation samples by down-sampling patients without sepsis to match the overall sepsis prevalence of the unfiltered BerlinICU cohort. Note, however, that in continuous evaluation, the prediction horizon also determines the number of time points labeled as positive. So, while patient-level prevalence is fixed across strategies, sample-level prevalence varies with horizon in the continuous evaluation approach. As a result, prevalence-sensitive metrics are difficult to compare directly in continuous evaluation across different horizons. We therefore report both raw values and “lifted” variants that are normalized by the horizon-specific prevalence, namely PPV and AUPRC divided by prevalence and recall measured at the threshold where precision equals twice the prevalence, which disentangle model performance from shifts in baseline risk. When reporting precision at a fixed recall, we estimate it from the precision-recall curve by linear interpolation at the target recall.

To estimate the variability of performance metrics, we applied bootstrap resampling (1000 samples). For each evaluation setting that included a prediction horizon, we only included those patients whose length of stay matched or exceeded the defined horizon (Table S2 in Multimedia Appendix 1).

### Calibration Assessment

To assess model calibration, we computed the Brier score. Internal calibration on the MIMIC-IV dataset is reported in our previous publication [17]. For the external BerlinICU dataset, calibration was estimated using 5-fold cross-validation. In each fold, isotonic regression was fitted on 80% of the data and applied to the held-out 20% to produce calibrated predictions. All performance metrics were calculated using the uncalibrated model applied to the full BerlinICU dataset, since discrimination metrics such as AUROC, precision, or recall are unaffected by probability calibration and depend only on the ranking of predicted scores, which is not changed by calibration. Calibration analyses were performed using scikit-learn (version 1.7.0).

### Clinical Utility

Although the primary objective of this study was to compare evaluation strategies, we included a clinical utility analysis with a continuous evaluation setup, which best aligns with clinical reality. Intensive care early-warning systems generate continuously updated risk estimates that may trigger alerts throughout the patient’s stay rather than single patient-level decisions. We quantified how the model would behave when deployed as an hourly alerting system by identifying a threshold corresponding to 80% sensitivity for sepsis onset and defining an alarm as any hourly prediction exceeding this threshold. Alarm rates for septic and nonseptic admissions were calculated as the mean number of alarms per patient-hour. To approximate typical alert-suppression logic, we applied a 4-hour silencing window; after an alarm was triggered for a patient, subsequent alarms for that patient were suppressed for 4 hours, and alarm rates were recomputed using only unsilenced alerts. Net benefit was evaluated using decision-curve analysis implemented with the dcurves package, where net benefit represents the proportion of true-positive alerts minus a threshold-dependent penalty for false-positive alerts. For the main clinical interpretation, we focus on the continuous evaluation with a 6-hour prediction horizon, but also report net benefits for the other strategies to illustrate how the choice of evaluation strategy influences apparent clinical usefulness.

### Statistical Analysis

To assess the significance of AUROC differences between evaluation strategies, we used a paired bootstrap Wald test with 1000 bootstrap samples per strategy. For each comparison, we drew the same bootstrap sample of patients for both strategies, computed AUROC for each, and formed the per-replicate difference. The SE of the difference was estimated as the SD of the bootstrap differences. Furthermore, 2-sided *P* values were obtained from the standard normal distribution. We applied multiple comparison correction at the level of hypothesis families; for the onset-matching effect within each strategy×model×dataset there is exactly one prespecified contrast (with vs without onset), whereas for strategy comparisons, we tested the 3 related pairwise contrasts (fixed vs peak, peak vs continuous, and fixed vs continuous) within each model×dataset×onset condition. To control the probability of at least 1 false positive among these related tests, we used the Holm step-down procedure.

## Results

### Study Cohort

**Figure 3:**
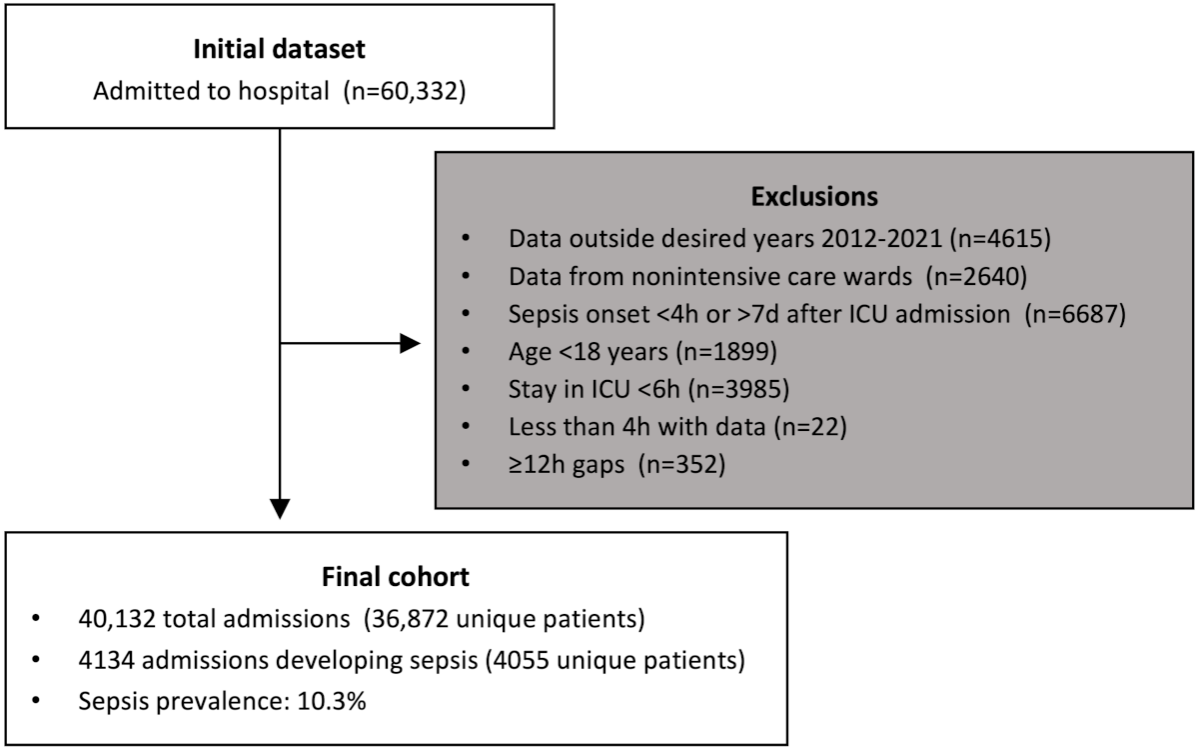
Flow diagram of patient eligibility criteria. ICU: intensive care unit.

From 60,332 ICU stays in the initial dataset, after extraction, preprocessing, and filtering, the final dataset consisted of 40,132 ICU stays of 36,872 unique patients spanning 10 years (2012-2021). Furthermore, 4134 stays were septic, resulting in a sepsis prevalence of 10.3% (Figure 3, Table 1). The original MIMIC-IV training dataset consisted of 67,056 ICU stays with a sepsis prevalence of 5.6% (3730 ICU stays).

**Table 1:** Patient characteristics of BerlinICU and MIMIC-IV^a^ for the total dataset, the sepsis cohort, and nonsepsis cohort.

Statistic			Total	Sepsis	Control
**BerlinICU**					
	Cohort size n, (row-%)		40,132 (100)	4134 (10)	35,998 (90)
	Age (y), median (IQR)		64 (50-75)	67 (55-77)	63 (50-75)
	ICU^b^ length of stay (d), median (IQR)		1.0 (0.7-2.1)	2.0 (1.4-3.4)	1.0 (0.7-2.0)
	**Sex, n (%)**				
		Male	22,423 (56)	2444 (59)	19,979 (56)
		Female	17,709 (44)	1690 (41)	16,019 (44)
	**Patient count,** **n (%)**				
		Patients on vasopressors	17,117 (43)	3442 (83)	13,675 (38)
		Patients ventilated	13,967 (35)	2774 (67)	11,193 (31)
		Patients on any antibiotics	22,155 (55)	4134 (100)	18,021 (50)
		Patients on antibiotics for ≥3 days	5351 (13)	4134 (100)	1217 (3)
**MIMIC-IV**					
	Cohort size, n (row-%)		67,056 (100)	3730 (5.6)	63,326 (94.4)
	Age (y), median (IQR)		65 (53-76)	67 (54-76)	65 (53-76)
	ICU length of stay (d), median (IQR)		1.7 (1.0-2.8)	0.8 (0.6-1.5)	1.7 (1.0-2.8)
	**Sex, n (%)**				
		Male	37,184 (55)	2132 (57)	35,052 (55)
		Female	29,872 (45)	1598 (43)	28,274 (45)
	**Patient count,** **n (%)**				
		Patients on vasopressors	8548 (13)	1230 (33)	7318 (12)
		Patients ventilated	21,863 (33)	2520 (68)	19,343 (31)
		Patients on any antibiotics	46,276 (69)	3730 (100)	42,546 (67)
		Patients on antibiotics for ≥3 days	6214 (9)	2760 (74)	3454 (5)

MIMIC-IV: Medical Information Mart for Intensive Care IV.

ICU: intensive care unit.

### Calibration

In the external BerlinICU dataset, the TCN model’s predicted probabilities were reasonably calibrated, with a Brier score of 0.04-0.09 depending on the evaluation strategy (Figure S4 in Multimedia Appendix 1). Importantly, calibration does not affect the performance metrics reported in the main results, as these are independent of the predicted probability values themselves and are unaffected by monotonic transformations.

### Influence of Evaluation Strategy on Model Performance Estimates

**Figure 4:**
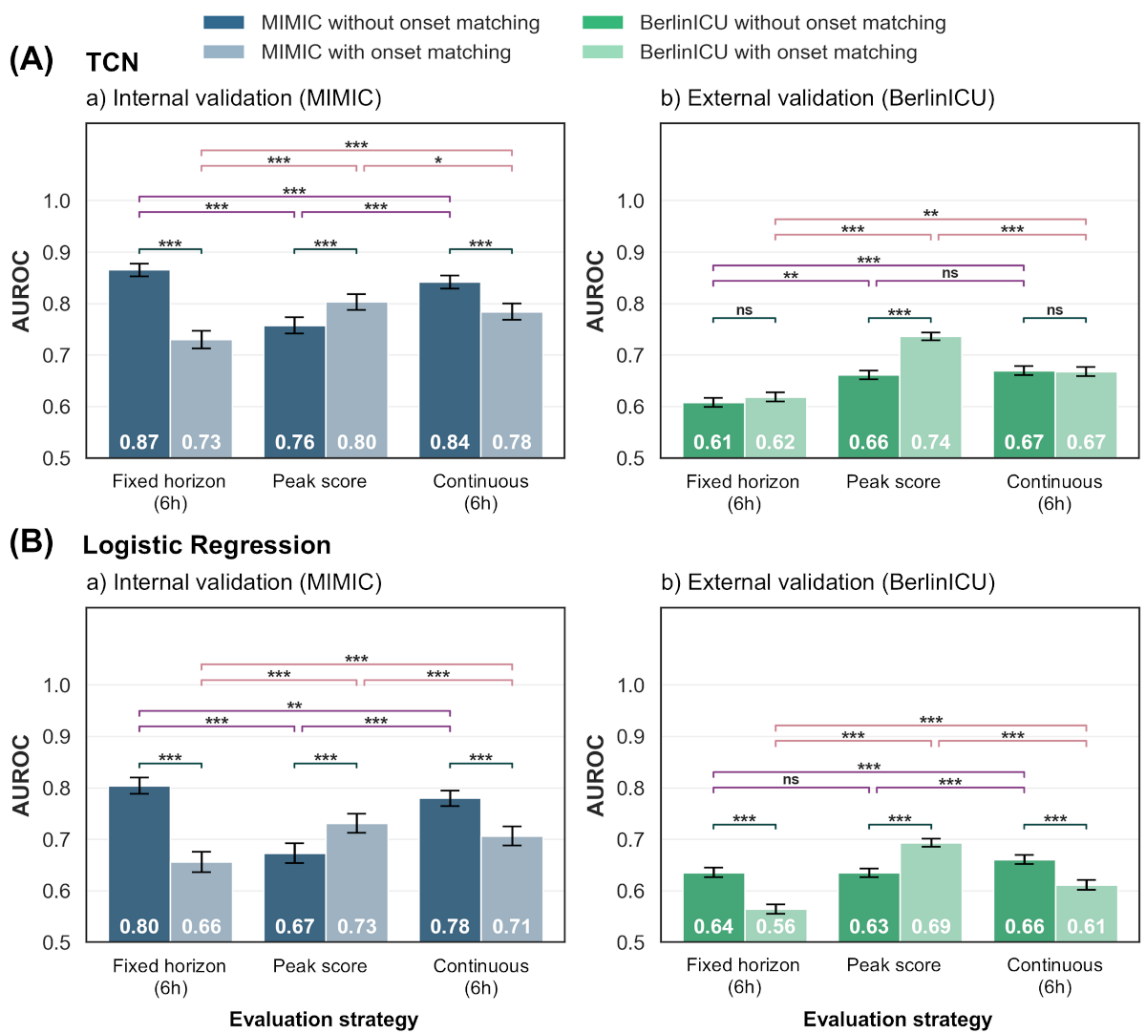
Comparison of model performance of (A) temporal convolutional network models (top) and (B) logistic regression baseline models (bottom) for different evaluation strategies. The models were applied to the test splits of the (a) MIMIC-IV training dataset and (b) the entire German ICU dataset (BerlinICU). Model performance was evaluated using fixed horizon evaluation (6h horizon), peak score evaluation (entire patient stay), and continuous evaluation (6h horizon) without onset matching (dark bars) and with onset matching (light bars). The AUROC value inside the bar represents the mean AUROC across all bootstrap samples and models. Error bars represent 95% CIs. Significance: ***P<.001, **P<.01, *P<.05, ns: not significant (paired bootstrap Wald test with Holm-adjusted *P* values). AUROC: area under the receiver operating characteristic curve; MIMIC: Medical Information Mart for Intensive Care.

As our primary analysis, we applied the TCN models pretrained on MIMIC-IV to the MIMIC-IV test set for internal validation under a scenario aligned with the training setup—continuous evaluation using a 6-hour prediction horizon without onset matching. Under this configuration, the models showed strong internal validation performance (Figure 4A). When the same models were applied to BerlinICU for external validation under the same evaluation conditions, performance was notably reduced (Figure 4B).

To assess the impact of different evaluation strategies, we performed fixed horizon evaluation under the same conditions (6-hour prediction horizon, no onset matching) and peak score evaluation, which does not use a prediction horizon. On the MIMIC-IV test set, fixed horizon evaluation resulted in a slightly higher AUROC, whereas peak score evaluation showed reduced performance. In external validation on BerlinICU, fixed horizon evaluation was markedly lower than continuous evaluation, while the peak score evaluation estimate was similar to that of the continuous evaluation. Across all evaluation strategies, performance estimates were consistently lower on BerlinICU compared with the MIMIC-IV test set.

Aligning the length of stay distribution of control patients to patients with sepsis through onset matching led to a notable AUROC reduction in internal validation on the MIMIC-IV test set for continuous evaluation (ΔAUROC=–0.06) and fixed horizon evaluation (ΔAUROC=–0.14), while AUROC estimates for peak score evaluation increased (ΔAUROC=0.04). On BerlinICU, onset matching had minimal impact on performance estimates during continuous evaluation or fixed horizon evaluation. In contrast, for peak score evaluation, onset matching increased AUROC estimates (ΔAUROC=0.08).

Across all evaluation strategies, TCN models consistently outperformed logistic regression baseline models. The only exception was observed in fixed horizon evaluation on BerlinICU without onset matching, where logistic regression showed a superior performance compared with TCN.

### Influence of the Prediction Horizon on Model Performance Estimates

**Figure 5:**
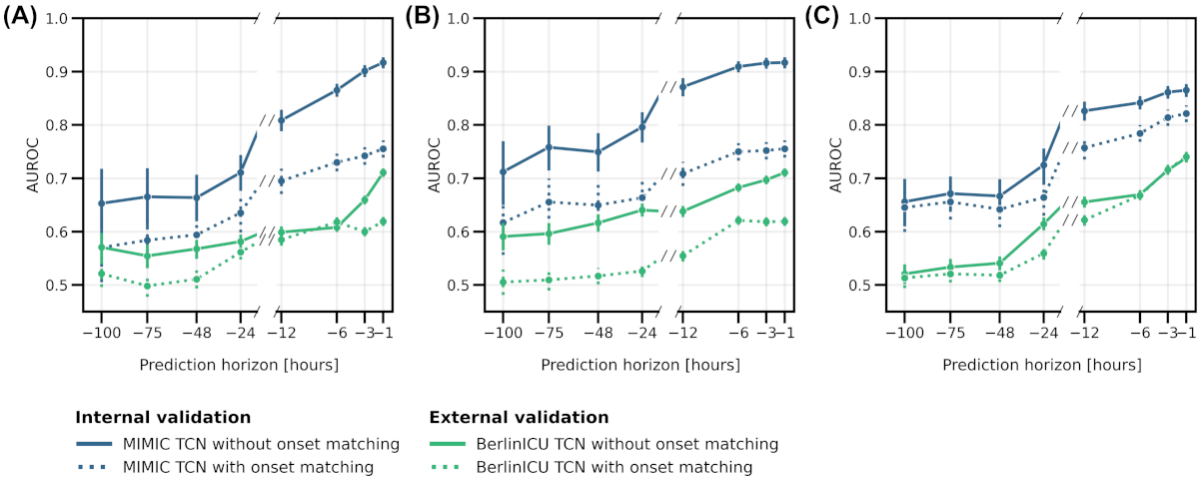
Performance metrics across prediction horizons, comparing the performance of temporal convolutional network models for an evaluation with onset matching (dashed lines) and without onset matching (solid lines). (A) Fixed horizon, (B) peak score, and (C) continuous prediction. The models were applied to the test splits of the MIMIC-IV training dataset (blue) and the entire German ICU dataset (BerlinICU; green). Shown is the AUROC based on varying prediction horizons from one hour before onset up to 100 hours before onset. Error bars represent 95% CIs. AUROC: area under the receiver operating characteristic; MIMIC: Medical Information Mart for Intensive Care; TCN: temporal convolutional network.

To evaluate the ability of the models to provide timely predictions, we assessed their performance across a range of prediction horizons (Figure 5). Performance estimates generally improved with shorter prediction horizons across all evaluation strategies. During internal validation on the MIMIC-IV test set, fixed horizon evaluation and continuous evaluation showed consistent improvement, with the highest AUROC achieved at a 1-hour horizon (fixed horizon: AUROC=0.92, 95% CI 0.91-0.93; continuous: AUROC=0.87, 95% CI 0.85-0.88). Peak score plateaued around the 6-hour horizon (AUROC 0.92, 95% CI 0.91-0.93), with only marginal improvements for smaller horizons. In external validation on BerlinICU, performance also generally increased with shorter prediction windows, with few exceptions, including a plateau around the 6-hour horizon in fixed horizon and peak score evaluation with onset matching.

Onset matching consistently decreased performance estimates during internal validation on the MIMIC-IV test set across all evaluation strategies, albeit to different extents, with continuous evaluation showing the smallest susceptibility to the matching approach. On BerlinICU, onset matching impacted strategies differently depending on the prediction horizon. Notably, continuous evaluation remained more robust to onset matching, maintaining more consistent AUROCs compared with the other strategies across different prediction horizons, particularly for very short and long prediction horizons.

Comparing the TCN and logistic regression baseline models in internal validation on the MIMIC-IV test set, TCN models consistently outperformed logistic regression models across all evaluation strategies and prediction horizons. In contrast, during external validation on BerlinICU without onset matching, logistic regression models approached or even exceeded the performance of TCN models at certain prediction horizons (Figure S1 in Multimedia Appendix 1). Onset matching was associated with an AUROC reduction for both models; the reduction appeared larger for logistic regression than for TCN in our data.

### Clinical Operational Characteristics Under Continuous Evaluation

Under the continuous evaluation with a 6-hour prediction horizon, an operating point of 80% sensitivity produced 0.70 alarms/hour in patients with sepsis and 0.25 alarms/hour in patients without sepsis in MIMIC-IV, compared with 0.74 and 0.55 alarms/hour, respectively, in BerlinICU (Figure S8 in Multimedia Appendix 1). With a 4-hour silencing period, rates decreased to 0.18 (septic) and 0.06 (nonseptic) in MIMIC-IV, and to 0.18 and 0.14 alarms/hour, respectively, in BerlinICU. Decision-curve analysis showed modest net benefit at low thresholds in MIMIC-IV and limited benefit in BerlinICU (Figure S9 in Multimedia Appendix 1).

## Discussion

### Principal Findings

In this study, we assessed the impact of commonly used evaluation strategies (fixed horizon, peak score, and continuous evaluation) on the performance of machine learning models for sepsis onset prediction. Our results demonstrate that the choice of the evaluation strategy significantly influences performance estimates, even though the same model is used on the same dataset.

We further addressed a gap in research involving the German health care system by aggregating an ICU dataset, called BerlinICU, from one of the largest university hospitals in Europe, Charité - Universitätsmedizin Berlin. Including more than 40,000 admissions from 8 ICU wards, this dataset is one of the largest ICU datasets in Europe, exceeding the size of the currently available European datasets—Amsterdam University Medical Center Database [31], High Time Resolution ICU Dataset [32], and Salzburg Intensive Care database [33]. To the best of our knowledge, this is one of the first studies to explore how sepsis prediction models perform on large-scale German ICU data.

### Fixed Horizon Evaluation Requires Prospective Knowledge of Sepsis Onset

Fixed horizon evaluation uses data up to a predefined time point before sepsis onset—the prediction horizon—to make predictions. Performance decreased with longer prediction horizons due to increasing temporal distance from sepsis onset. Without onset matching, performance estimates strongly increased for all horizons on the MIMIC-IV test set and for most horizons for BerlinICU, with the exception of the 6-hour horizon. This performance increase when not using onset matching is most parsimoniously explained by the evaluation of control patients over their entire stay, with emphasis on the period just before discharge. During these predischarge hours, control patients are typically in a stable condition, making it easier for the model to correctly identify them as low-risk for sepsis. This effect, also noted by Futoma et al [29], shows the significant impact of onset alignment. Despite this significant influence, many studies do not comment on the implementation of case-control matching [13]. While the fixed horizon approach achieved the highest AUROC in a study on different evaluation strategies [19], we observed more nuanced performance differences compared with the other evaluation approaches, depending on the dataset, horizon, and onset matching approach. Fixed-horizon evaluation can be useful for benchmarking how far in advance a model can predict sepsis and for retrospective simulation studies, but its direct clinical interpretability is challenging, as it relies on prospective information—the time point of the sepsis onset—which is not accessible in a real-world clinical setting.

### Peak Score Evaluation is Influenced by the Length of Stay Distribution and Requires Onset Matching to Avoid Bias

The peak score evaluation assesses the model’s peak confidence in predicting sepsis throughout the entire ICU stay. This approach seems intuitively aligned with clinical practice, where a fixed threshold would be predetermined, and an alarm is triggered once the prediction model’s output surpasses it—potentially just once per patient, with subsequent alarms suppressed [16,20]. The focus is on whether an alarm is raised at any point during the patient’s stay, which is replicated by determining the maximum prediction score. However, the performance metrics determined under this approach are influenced by differences in length of stay distributions between cases and controls—longer time series for control patients increase the likelihood of higher maximum prediction scores for these patients simply due to random fluctuations, ultimately reducing the AUROC as the maximum scores for control patients approach those of patients with sepsis on average (Figure S3 in Multimedia Appendix 1). Importantly, this bias conflicts with the core interpretation of the AUROC, which measures the probability that a randomly selected true positive will have a higher predicted score than a randomly selected true negative. Our results demonstrate this behavior, showing that performance estimates without onset matching, where control cases tend to have longer lengths of stays, were significantly reduced compared with analyses with onset matching (Figure 4). To mitigate this effect, an onset matching approach that aligns the length of stay distributions between positive and negative cases is strictly necessary when applying this evaluation strategy.

The discrepancy between the peak score evaluation across all patients (Figure 4), where onset matching increases the AUROC, and horizon-dependent analysis (Figure 5), where onset matching decreases the AUROC, can be explained by the exclusion of patients with insufficient stay lengths in the latter. This exclusion alters the length of stay distribution and thereby significantly impacts performance metrics. Not filtering patients with insufficient length of stays shows a distinct interaction effect between onset matching and prediction horizon (Figure S2 in Multimedia Appendix 1), further highlighting the critical influence of length of stay distribution on the performance estimates in the peak score evaluation approach. Nevertheless, peak score evaluation has a simple clinical interpretation—at a given threshold, does the model ever raise an alarm for a patient with sepsis? When the length of stay distribution is carefully adjusted between cases and controls and the evaluation is restricted to a clinically meaningful time frame (eg, within 12 hours before sepsis onset), this strategy can provide a useful summary of whether the model is able to flag a patient at high-risk at any point during their stay.

### Continuous Evaluation Without Onset Matching Reflects Clinical Practice

Continuous prediction evaluation showed increased performance with shorter prediction windows, demonstrating the model’s effectiveness in using short-term, relevant data to predict sepsis onset. As the prediction window narrows and focuses more on the actual onset of sepsis, the assigned sepsis labels align more closely with the sepsis-related signals in the patient data, enhancing prediction efficiency. Interestingly, although the models were trained to predict sepsis 6 hours in advance, this specific labeling window did not yield the best performance. This is likely because these shorter windows include only time points immediately before sepsis onset, where the signal is strongest, while earlier time points, although possibly already showing signs of deterioration, are treated as negatives.

Evaluating on BerlinICU with onset matching, model performance was significantly reduced compared with the MIMIC-IV test set (∆AUROC=–0.11 for 6 hours before onset and –0.08 for 1 hour). This reduced performance when transferring models between datasets was also evident in our previous study, where models trained on MIMIC-IV were evaluated on other European datasets using a horizon-based evaluation with a 6-hour window [17]. Even though the performance in that study declined for European datasets (High time Resolution ICU Dataset: ∆AUROC=–0.07, Amsterdam University Medical Center Database: ∆AUROC=–0.09) as well as for another US dataset (Electronic Intensive Care Unit Collaborative Research Database: ∆AUROC=–0.10), it was not as severe as observed in BerlinICU. One might suspect that this sharper decline is related to different sepsis prevalences, since BerlinICU had a higher prevalence than MIMIC-IV. However, AUROC is unaffected by prevalence [21], and the difficulty of the task, and therefore the prediction performance, is more strongly influenced by factors such as data heterogeneity, label noise, and shifting feature distributions, all of which may differ substantially between datasets. These findings underscore the importance of addressing domain shift when deploying machine learning models across health care systems. Differences in patient populations, documentation practices, feature availability, and underlying sepsis definitions may contribute to reduced generalizability. For example, differences in clinical workflows, such as the timing and frequency of laboratory testing, documentation delays, or variability in when and how sepsis-related interventions are initiated (eg, antibiotics or fluids), may influence how early or clearly sepsis-related signals appear in the data. The use of missingness indicators is another potential factor, although previous studies have reported mixed effects on the generalizability of sepsis prediction models [22,28]. Mitigation strategies, such as dataset-specific fine-tuning or full retraining on local data, can be considered to improve cross-domain performance and support safe clinical deployment.

Without onset matching, performance in the MIMIC-IV test set was higher across horizons, and was similar or better on BerlinICU for most horizons. Again, this higher performance without onset matching most likely reflects the inclusion of time points closer to discharge for control patients, who generally have a healthier profile, making the prediction task easier. This mechanism is illustrated in Figure S10 in Multimedia Appendix 1, which shows predicted risk trajectories over time; unmatched controls display a marked decline in predicted risk approaching discharge, whereas matched controls remain at higher levels, closer to those of patients with sepsis. While onset matching may provide a focused assessment in more challenging cases, not using it better mirrors clinical practice, where patients are monitored continuously from admission to discharge. In our view, continuous evaluation without onset matching better reflects real-world clinical application, as the AUROC represents the likelihood of a positive case being ranked higher than a negative one, including time points closer to discharge. Accordingly, we treat continuous evaluation without onset matching as the most clinically relevant interpretation of model performance, while the other evaluation strategies are used to demonstrate how alternative designs can substantially alter reported results.

Although this approach requires predetermining a time window, which indicates how far in the future the onset may be predicted, we consider continuous evaluation the better choice for 2 reasons. First, defining a time window—ideally based on pathophysiological plausibility—ensures that only alarms occurring within a clinically meaningful period before sepsis onset are considered. This distinction is crucial, as some published models show high performance days to weeks before the actual event [18,34,35], likely detecting broader, correlated health-related features rather than sepsis specifically. This underlines the importance of thoroughly defining and testing what these models are predicting to ensure their practical utility in clinical settings. Second, varying the prediction window and reporting the performance across all these windows allows for a more nuanced understanding of the models’ ability to predict event onset in advance. This analysis can be performed efficiently without retraining, since the underlying model predictions remain fixed and only the labeling of time points is varied to reflect different prediction windows. However, training separate models for different forecast horizons and investigating the effect on the performance using continuous evaluation is an interesting, but computationally more intensive approach that could be explored in future studies.

It is important to note that longer prediction horizons (eg, beyond 12 h) are unlikely to be clinically meaningful. From a clinical standpoint, alarms triggered more than half a day before sepsis onset may not prompt specific interventions and could increase alert fatigue. Nevertheless, we included prediction horizons up to 100 hours as a form of negative control to investigate whether model performance persists far in advance of sepsis. If so, this could indicate that the model is capturing nonspecific markers of deterioration or health status rather than sepsis-specific signals. In line with this, we observed that the model’s performance on BerlinICU dropped toward chance level at long horizons, while AUROC remained elevated on MIMIC-IV, possibly suggesting that the model may overfit to nonspecific or site-specific patterns in the training data.

### Clinical Implications of the Choice of the Evaluation Strategy

The evaluation strategy directly determines how performance metrics translate to clinical practice. Metrics such as PPV, when derived from peak-score evaluation, are patient-level; they yield a single value per patient that answers, “Did this patient ever cross the alert threshold?” In routine deployment, however, models are usually executed repeatedly (eg, hourly), and clinicians need alert-level information, that is, the probability that a specific alarm corresponds to true sepsis within a clinically relevant time window. Using patient-level metrics to select models for clinical deployment can therefore overestimate real-world performance and contribute to alarm fatigue. Likewise, fixed-horizon evaluation reports performance at one predefined time point per patient; it does not describe how the model behaves during continuous monitoring. Importantly, it requires prospective knowledge of the exact onset time, which is inherently unavailable in clinical reality. For continuous evaluation, the horizon is adjustable; PPV at *h* hours answers the question “If the model raises an alert now, what is the probability that sepsis will occur within the next h hours?” Different choices of the horizon yield different PPVs, and only the PPV at the clinically intended horizon is directly relevant for deployment. Thus, to ensure clinical relevance, evaluation strategies and metrics should be aligned with how the model will be used in practice.

Our analyses focused primarily on TCN models. However, the effects of the evaluation strategy are not specific to this architecture. Because evaluation is applied post hoc to model outputs, the principles are, in theory, model-agnostic. Consistent with this expectation, we observed the same qualitative influence of evaluation strategy on performance when applying it to GRU- and transformer-based models (Figure S7 in Multimedia Appendix 1). This supports the interpretation that our findings reflect general properties of evaluation design rather than model-specific behavior. Likewise, while our empirical analyses were conducted on 2 datasets, the observed differences between evaluation strategies arise from the way labels are constructed and metrics are defined, and are therefore expected to hold independently of the specific dataset.

### Clinical Utility Considerations

While the primary objective of this study is to compare evaluation strategies, it is helpful to illustrate how a model of this type would function within a real-time ICU workflow. In practice, such early-warning systems would generate repeated risk estimates and trigger alerts that prompt targeted clinical assessment, such as reviewing chart data or performing additional tests, rather than automated interventions. Thresholds are typically chosen to balance timely detection with acceptable alarm burden, often combined with silencing periods to avoid repeated notifications. Since clinical use is structured around continuous monitoring and repeated predictions, continuous evaluation is the strategy that most closely reflects real-world deployment.

The operational characteristics observed in our analyses provide an indication of the potential alarm burden such a system might generate. At 80% sensitivity, the model produced alarm rates that are plausible for an ICU early-warning context, although their clinical acceptability is uncertain and would require prospective evaluation. A 4-hour silencing period substantially reduced repeated alerts, highlighting the importance of workflow-oriented design choices. Decision-curve analysis further showed modest net benefit at low thresholds in MIMIC-IV and limited benefit in BerlinICU.

### Limitations

Our study has several limitations. First, we did not have access to microbiological data, requiring an adjustment of the Sepsis-3 definition. While this approach has been used in previous studies using hospital datasets without microbiology records [16,17], it may reduce the specificity of outcome labeling and limit comparability with studies that apply the complete Sepsis-3 criteria, including microbiology data. Second, we used models trained for a 6-hour horizon and applied them across various prediction horizons to predict sepsis onset. While these models may not perform optimally outside their original specifications, the inability to know the exact duration until a potential sepsis onset at any given timepoint in clinical practice necessitates using a flexible approach. Thus, by testing these models beyond their intended parameters, we can evaluate their robustness and generalizability to different clinical scenarios and timelines. We acknowledge, however, that this does not replace training and comparing separate models optimized for different prediction horizons.

### Comparison With Previous Work

Our work combines the idea of generalizability across countries [16,17] and suitable metric calculation concepts [16,36]. Our study builds on these previous concepts, refining and extending them as needed. While Moor et al [16] and Rockenschaub et al [17] examined the influence of various algorithms and training sets from different countries, our work focuses on different evaluation strategies. We applied sepsis onset matching [29,36] and peak score evaluation [16], combining approaches that were presented separately. As the peak score evaluation is only suitable for retrospective analysis, we also applied a continuous evaluation strategy [13]. Moreover, there are only a few studies that have applied models to German data [18,37], none of which have a dedicated focus on comparing performances in different evaluation strategies. While some models have reported high AUROC values on German data, care is needed when interpreting or comparing such metrics across studies, as the AUROC is not a function of the model alone, but depends heavily on the prediction task and the underlying dataset. Differences in prediction target, patient population, and labeling criteria can have substantial effects on reported discrimination. For example, distinguishing patients with sepsis in the ICU from a general hospital population is inherently easier than distinguishing sepsis from nonsepsis within an already critically ill ICU cohort, due to the greater physiological overlap in the latter case. Moreover, as we demonstrate in this study, the choice of evaluation strategy (eg, fixed prediction horizon, onset matching, or continuous evaluation) can meaningfully influence reported performance.

Several studies have applied sepsis prediction models prospectively to US data [14,15,38], but they often lack details on how the models were trained and how the data were preprocessed. This lack of transparency is a common issue, as noted in 2 systematic reviews of sepsis prediction models [12,13]. Accordingly, common checklists for reporting prediction models include items for external validation in different countries and time periods [39,40].

### Conclusions

Our study highlights the critical need to choose an evaluation approach that is aligned with the intended interpretation of the resulting performance metric, as different approaches yield markedly different performance estimates despite using the same model on the same dataset. Importantly, for the fixed horizon and peak score evaluation approaches, a carefully devised onset matching strategy is crucial to avoid skewed results that may not reflect the true model performance. In our view, the continuous evaluation approach better reflects clinical reality. Despite requiring specifying the horizon time window, it offers insights into the model’s effectiveness in a clinical setting.

## Data Availability

The MIMIC-IV (Medical Information Mart for Intensive Care IV) dataset is publicly available for research purposes through PhysioNet upon completion of a data use agreement and required training [41,42]. The BerlinICU dataset used in this study cannot currently be shared outside the institution due to data protection regulations and institutional policies. Preprocessing and training code for the neural network models has been published by Rockenschaub et al [17], with the corresponding GitHub repository available at GitHub [43]. All analysis code specific to this study are also available [44].

## Appendix

Multimedia Appendix 1 Additional tables and figures.

## Abbreviations

AUPRC: area under the precision-recall curve
AUROC: area under the receiver operating characteristic curve
ICU: intensive care unit
GRU: gated recurrent unit
MIMIC-IV: Medical Information Mart for Intensive Care IV
PPV: positive predictive value
NPV: negative predictive value
SI: suspicion of infection
SOFA: Sequential Organ Failure Assessment
TCN: temporal convolutional network
