# Supplementary material for: The Impact of Evaluation Strategy on Sepsis Prediction Model Performance Metrics in Intensive Care Data: Retrospective Cohort Study
Source: J Med Internet Res. 2026 Mar 24;28:e72083. doi: 10.2196/72083 (PMC13058531; doi:10.2196/72083)
Supplement: Multimedia Appendix 1 [file jmir_v28i1e72083_app1.pdf]

Supplementary Appendix

Feature name	Unit
**Static variables**	
Age at hospital admission	Years
Gender	male/female
Patient height	cm
Patient weight	kg
**Vital signs**	
Blood pressure (systolic)	mmHg
Blood pressure (diastolic)	mmHg
Heart rate	beats per minute
Mean arterial pressure	mmHg
Oxygen saturation	%
Respiratory rate	breaths per minute
Temperature	°C
**Arterial blood gas analysis**	
Base excess	mmol/L
Bicarbonate	mmol/L
CO_2 partial pressure	mmHg
Lactate	mmol/L
O_2 partial pressure	mmHg
pH of blood	-
**Electrolytes**	
Calcium (total)	mg/dl
Calcium ionized	mmol/L
Chloride	mmol/L
Magnesium	mg/dL
Potassium	mmol/L
Phosphate	mg/dL
Sodium	mmol/L
**Blood Count**	
Band form neutrophils	%
Hemoglobin	g/dL
Lymphocytes	%
Mean cell hemoglobin	pg
Mean corpuscular hemoglobin concentration	%
Mean corpuscular volume	fL
Methemoglobin	%
Neutrophils	%
Platelets	1,000 / uL
White blood cells	1,000 / pL
**Others**	
Albumin	g/dl
Alkaline phosphatase	IU/L
Alanine aminotransferase	IU/L
Aspartate aminotransferase	IU/L
Bilirubin (total)	mg/dL
Bilirubin (direct)	mg/dl.
Blood urea nitrogen	mg/dl
Creatinine	mg/dl
Creatinine kinase	IU/L
Creatinine kinase MB	ng/mL
C-reactive protein	mg/L
Fibrinogen	mg/dl
Glucose	mg/dL
International normalized ratio (INR)	-
Partial thromboplastin time	sec
Troponin T	ng/mL
Fraction of inspired oxygen	%
Urine output	mL

Table S1: Clinical features used in the prediction models.

Supplementary Results

Dataset | Horizon	Mean Total Patients (Min-Max)	Mean Sepsis Patients (Min-Max)	Mean Prevalence (%) (Min-Max)
**MIMIC-IV (TCN)**			
Total	13,411 (13,411- 13,411)	747 (709-830)	5.6 (5.3-6.2)
-100	1,664 (1,616-1,724)	60 (45-69)	3.6 (2.8-4.2)
-75	2,716 (2,613-2,783)	101 (89-115)	3.7 (3.3-4.3)
-48	5,184 (5,036-5,246)	149 (132-179)	2.9 (2.6-3.5)
-24	9,934 (9,882-9,990)	225 (207-271)	2.3 (2.1-2.7)
-12	12,686 (12,644-12,719)	435 (395-500)	3.4 (3.1-4.0)
-6	13,411 (13,411- 13,411)	747 (709-830)	5.6 (5.3-6.2)
-3	13,411 (13,411- 13,411)	747 (709-830)	5.6 (5.3-6.2)
-2	13,411 (13,411- 13,411)	747 (709-830)	5.6 (5.3-6.2)
-1	13,411 (13,411- 13,411)	747 (709-830)	5.6 (5.3-6.2)
**MIMIC-IV (LogReg)**			
Total	13,411 (13,411-13,412)	746 (746-746)	5.6 (5.6-5.6)
-100	1,676 (1,647-1,716)	58 (45-67)	3.4 (2.7-3.9)
-75	2,727 (2,685-2,800)	96 (89-106)	3.5 (3.3-3.8)
-48	5,203 (5,136-5246)	146 (137-153)	2.8 (2.6 – 2.9)
-24	9,926 (9,857-9,984)	218 (204-231)	2.2 (2.0 - 2.3)
-12	12,683 (12,664-12,703)	431 (408-442)	3.4 (3.2-3.5)
-6	13,411 (13,411-13,412)	746 (746-746)	5.6 (5.6-5.6)
-3	13,411 (13,411-13,412)	746 (746-746)	5.6 (5.6-5.6)
-2	13,411 (13,411-13,412)	746 (746-746)	5.6 (5.6-5.6)
-1	13,411 (13,411-13,412)	746 (746-746)	5.6 (5.6-5.6)
**BerlinICU**			
Total	40,155	4,137	10.3
-100	4,357	504	11.6
-75	6,277	773	12.3
-48	9,897	1,242	12.6
-24	18079	2,121	11.7
-12	33,798	2,871	8.5
-6	40,132	4134	10.3
-3	40,132	4134	10.3
-1	40,132	4134	10.3

Table S2: Cohort sizes for the time-dependent evaluation. *Shown is the number of patients in each cohort and dataset after filtering for patients whose length of stay matches or exceeds the prediction horizon. For BerlinICU, the total cohort size refers to the complete dataset, while for MIMIC-IV the mean, min and max across all test sets is described (TCN: 10 test sets, LogReg: 5 test sets). TCN: Temporal Convolutional Network. LogReg: Logistic Regression.*

Architecture	*Learning rate*	*Weight decay*	*Batch size*	*Hidden dims*	*Layers*	*Kernel size*	*Heads*	*Dropout*
TCN	4.5×10^{-4}	1e-5	128	64	8	3	-	0.3
GRU	4.5×10^{-4}	1e-5	128	64	8	-	-	0.3
Attention	7.8×10^{-4}	1e-6	128	32	2	-	2	0.5

Table S3: Tuned hyperparameters for the neural network architectures. *"Hidden dims" refers to the number of hidden units per layer, "Layers" to the number of stacked layers, and "Kernel size" to the convolutional kernel width (for TCN/GRU). "Heads" applies only to the Attention model. Dropout denotes the probability of dropping units during training.*

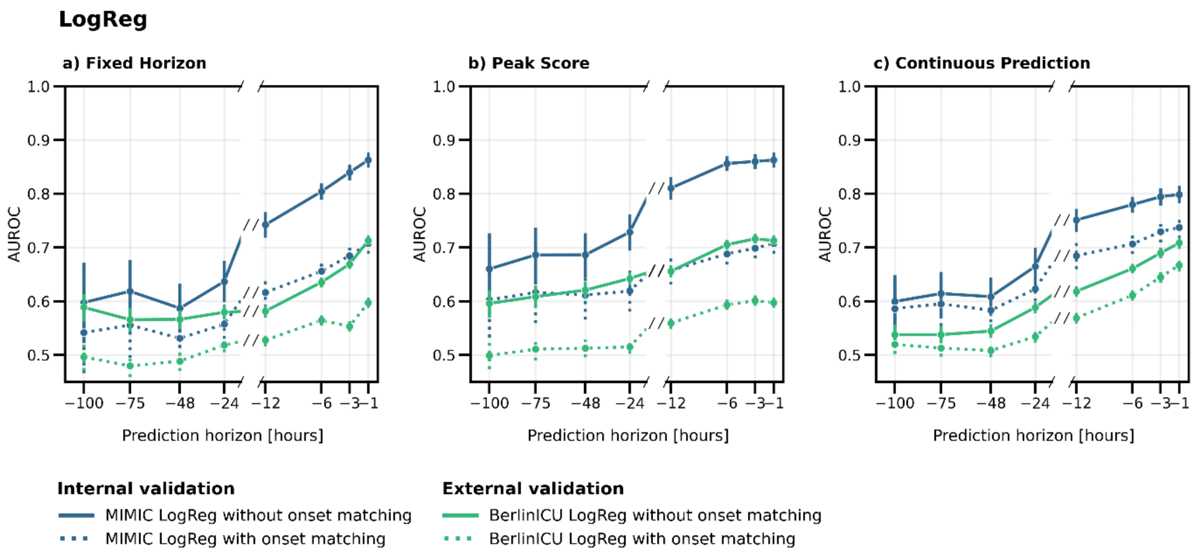

***Figure S1: Performance metrics across prediction horizons for logistic regression models**, comparing the performance an evaluation with onset matching (dashed lines) and without onset matching (solid lines). The models were applied to the test splits of the MIMIC-IV training dataset (blue) and the entire German ICU dataset (BerlinICU; green). Shown is the AUROC based on varying prediction horizons from one hour before onset up to 100 hours before onset. AUROC: area under the receiver operating characteristic. Error bars: 95% confidence intervals.*

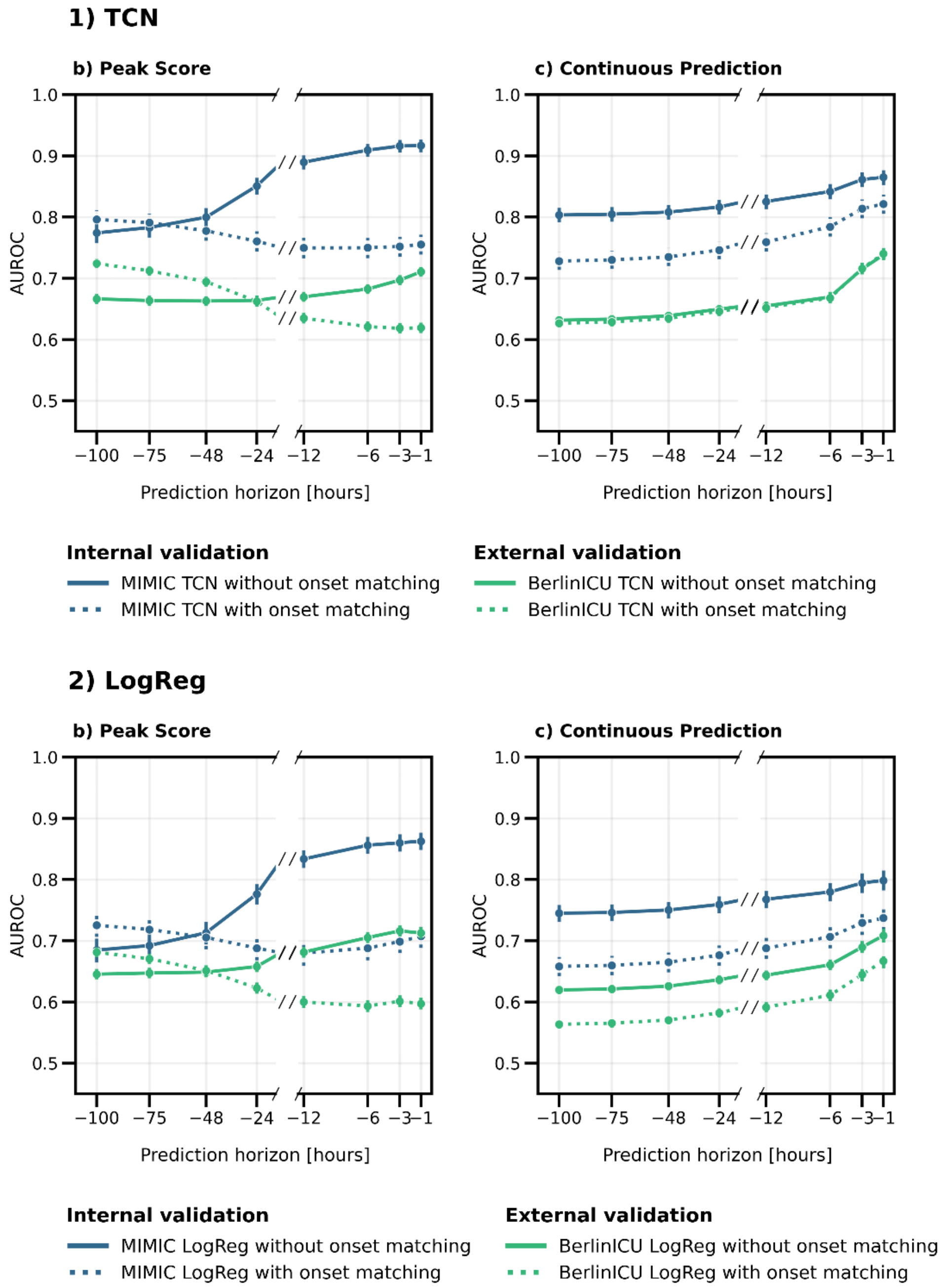

Figure S2: Performance metrics across prediction horizons, without filtering for length of stay (LOS), comparing, the performance of temporal convolutional network (TCN, a-b) and logistic regression (LogReg, c-d) models for an evaluation with onset matching (dashed lines) and without onset matching (solid lines). The models were applied to the test splits of the MIMIC-IV training dataset (blue) and the entire German ICU dataset (BerlinICU; green). Shown is the AUROC based on varying prediction horizons from one hour before onset up to 24 hours before onset. Each prediction horizon consisted of the same patients. If the LOS of a patient was shorter than the prediction horizon, then evaluation strategies were applied to the data points that were available. AUROC: area under the receiver operating characteristic. Error bars: 95% confidence intervals.

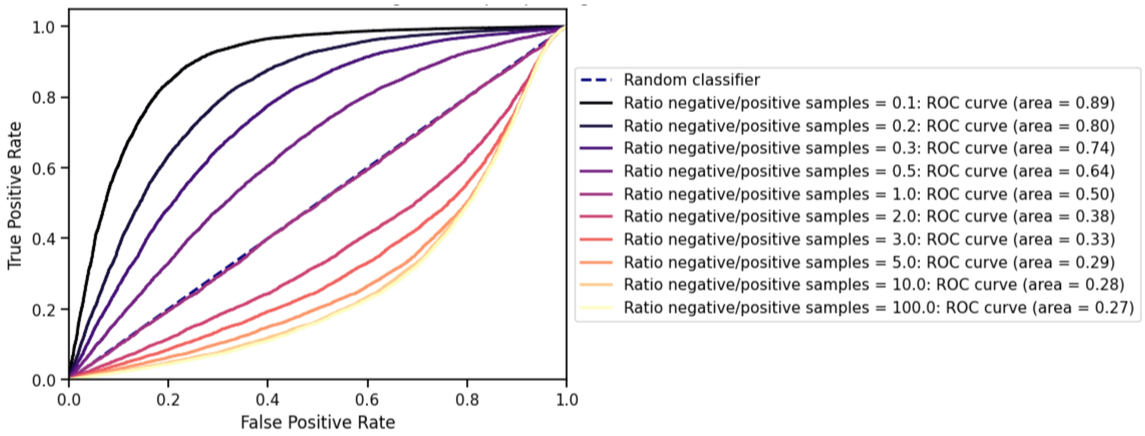

Figure S3: Peak Score Evaluation on Random Data with Increasing Maximum Number of Samples per Negative Patient. *The figure shows simulated ROC curves for a classifier trained on data in which there is no real difference between positive and negative patients, because the data for both groups are drawn from the same Gaussian distribution. Each group consists of 10,000 patients and each positive patient has a fixed number of 10 samples, while negative patients have varying numbers of samples, as indicated by the ratio in the legend. The ROC curves illustrate how differences in the number of samples per patient influence the maximum prediction score, artificially inflating or deflating performance metrics even though there is no true difference between the underlying data distributions for the two groups. Ratios below 1 (fewer negative samples; darker lines) artificially inflate performance, resulting in higher AUROC (>0.5). Ratios above 1 (more negative samples; lighter lines) lead to higher maximum scores for negative patients due to random fluctuations, reducing AUROC below 0.5. The diagonal line ("Random classifier") shows the baseline performance of a random classifier (AUROC = 0.5), which reflects the true expected performance given the absence of any signal or separability between the classes. This demonstrates that peak score evaluation can yield misleading performance estimates if the length of stay distributions differ significantly between patient groups, such as when length of stay differs between septic and non-septic patients.*

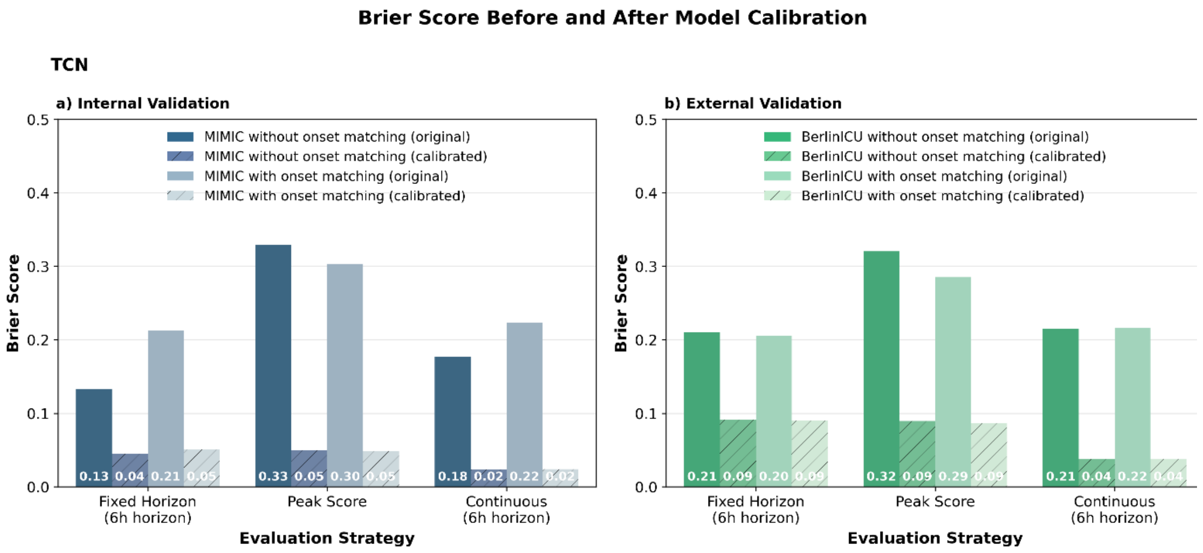

Figure S4: Comparison of Brier scores before and after calibration for the Temporal Convolutional Network (TCN) model under different evaluation strategies. *Panel (a) shows internal validation on MIMIC-IV data, and panel (b) shows external validation on BerlinICU data. Bars indicate the Brier score for the original models before calibration (solid fill) and the calibrated models (hatched fill) with (lighter color) and without (darker color) the inclusion of onset masking. Lower values indicate better calibration and overall prediction quality.*

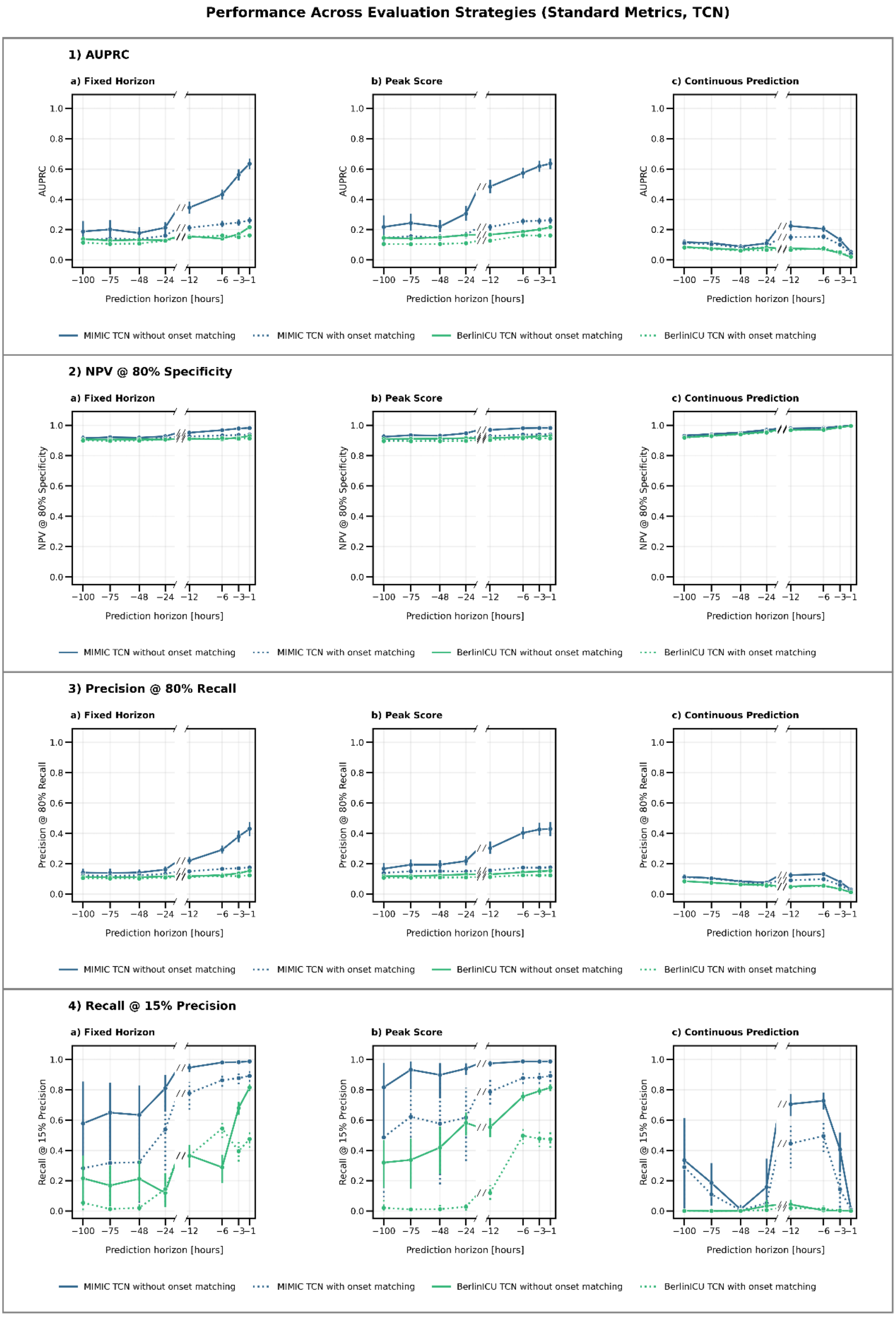

Figure S5: Performance across evaluation strategies (columns) and metrics (rows). *Columns show Fixed-horizon, Peak-score, and Continuous evaluation strategies. Rows show (1) Area under the Precision-Recall-Curve (AUPRC), (2) Negative Predictive Value (NPV) at 80% specificity, (3) Positive Predictive Value (PPV) at 80% recall, and (4) Recall at 15% PPV. Results are computed on time-point labels. Note that because for continuous evaluation, the horizon changes time-point prevalence, raw PPV decreases at shorter horizons even when discrimination is unchanged (see Figure S6 for prevalence-adjusted metrics).*

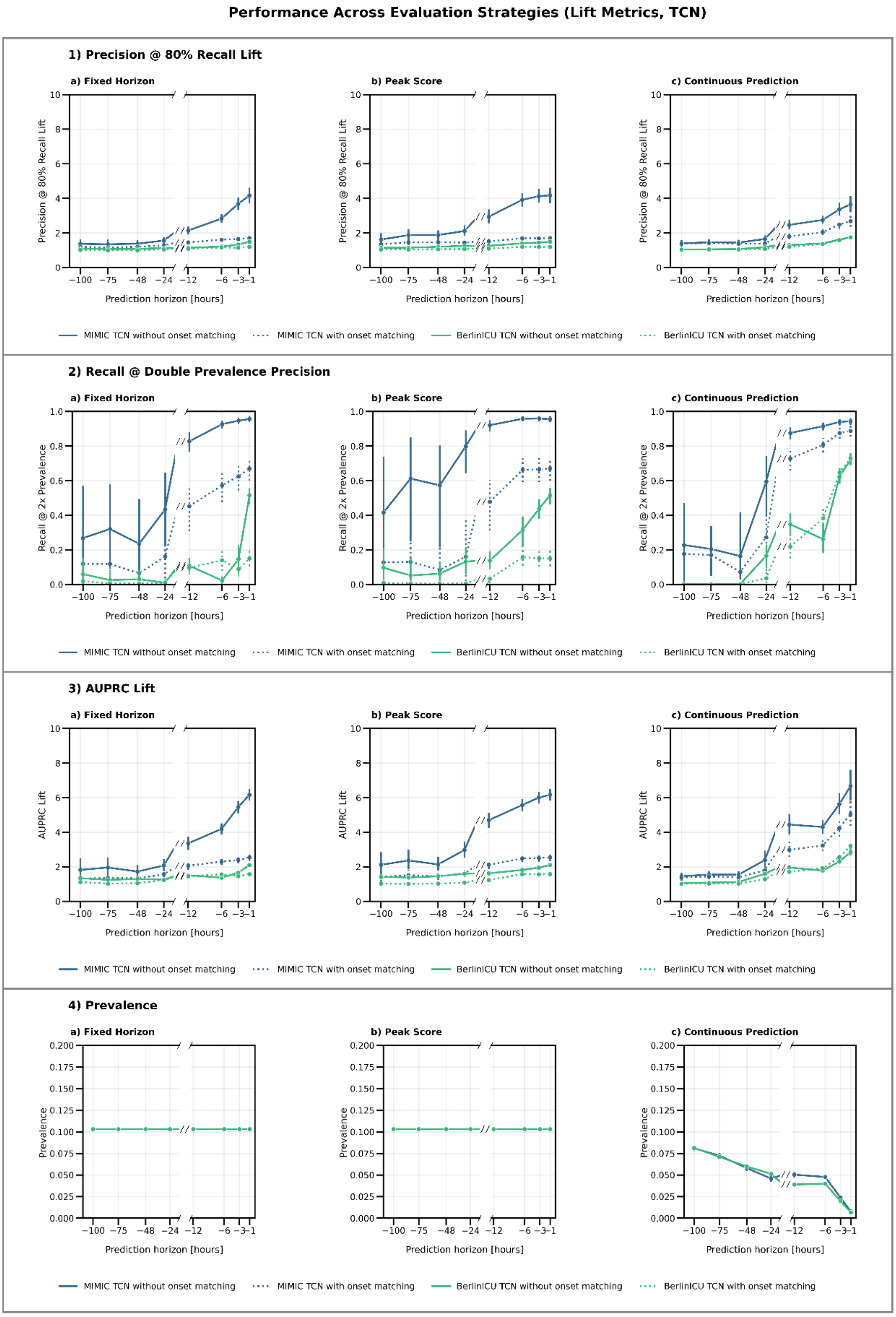

Figure S6: Lift-normalized metrics to account for horizon-specific prevalence*. (1) Positive Predictive Value (PPV) divided by prevalence at 80% recall (Precision @ 80% Recall Lift) quantifies how many-times-baseline the alerts are. (2) Recall at 2× prevalence replaces a fixed PPV target with a constant lift constraint. (3) Area under the Precision-Recall Curve (AUPRC) divided by prevalence (AUPRC Lift) quantifies improvement over baseline performance. (4) Prevalence is shown for different evaluation strategies and demonstrates how the prevalence in continuous prediction changes for each horizon. Lifted views complement the raw plots by separating model signal from changes in baseline event probability across horizons.*

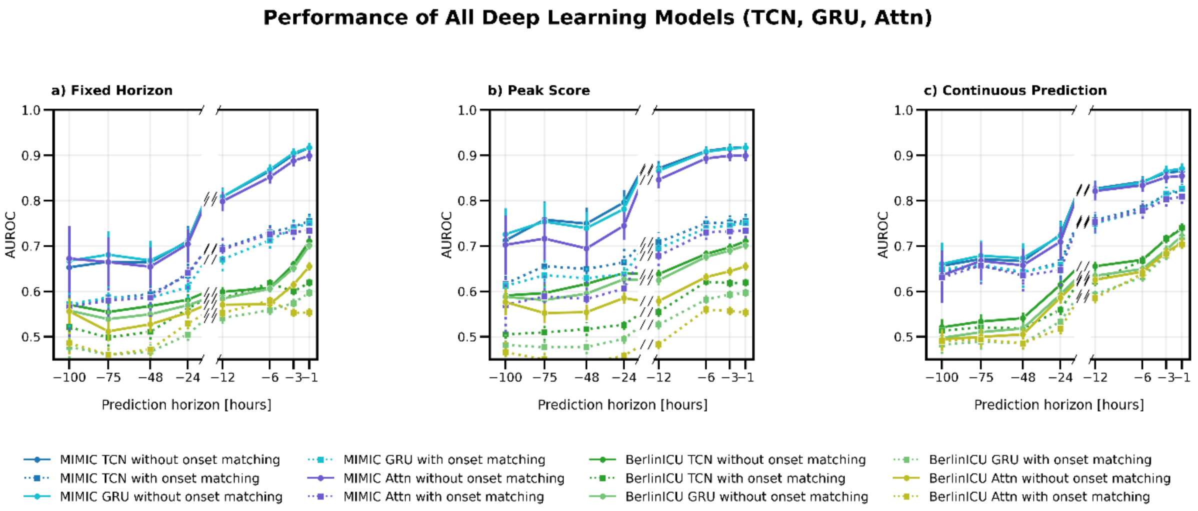

Figure S7: Performance metrics across prediction horizons for different neural network model architecture*, comparing the performance an evaluation with onset matching (dashed lines) and without onset matching (solid lines). The Temporal Convolutional Network (TCN), Gated-recurrent unit (GRU) and Attention-based (Attn) models were applied to the test splits of the MIMIC-IV training dataset and the entire German ICU dataset (BerlinICU). Shown is the Area under the Receiver-Operating Characteristic Curve (AUROC) based on varying prediction horizons from one hour before onset up to 100 hours before onset. AUROC: area under the receiver operating characteristic. Error bars: 95% confidence intervals.*

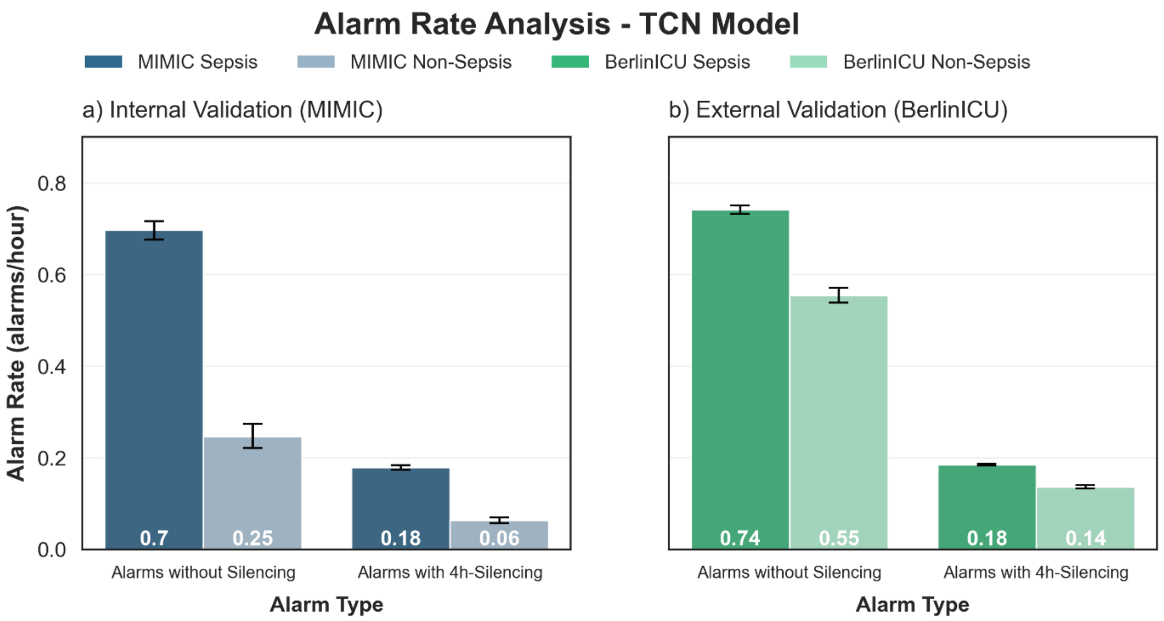

Figure S8: Alarm rate analysis under continuous evaluation. *Shown are the alarm rates for the temporal convolutional network (TCN) models under continuous evaluation with a 6-hour prediction horizon, evaluated on the test splits of the training dataset MIMIC-IV (a) and externally on BerlinICU (b). Bars display the mean number of alarms per patient-hour for septic and non-septic admissions at a decision threshold corresponding to 80% sensitivity. "Alarms without Silencing" counts every time point where the predicted risk exceeds the threshold; "Alarms with 4h-Silencing" applies a 4-hour alarm silencing after each alarm for the same patient. Numeric labels on the bars give mean alarm rates per hour; error bars indicate 95% confidence intervals.*

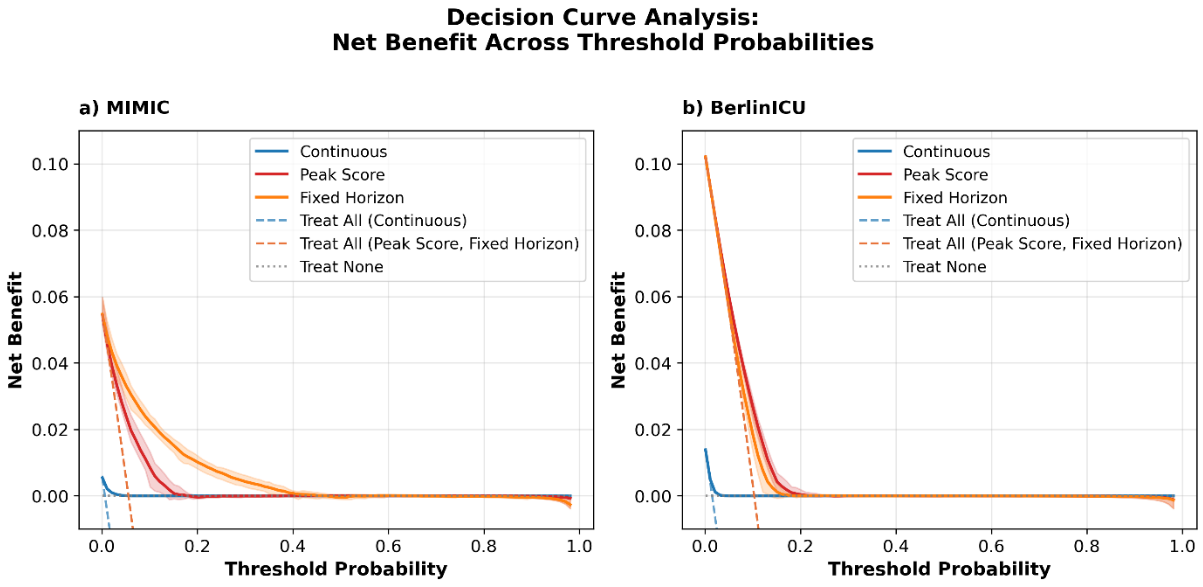

***Figure S9: Decision curve analysis under continuous evaluation**. Shown is the net benefit for the TCN sepsis model under different evaluation strategies for the test splits of MIMIC-IV (a) and external validation on BerlinICU (b). Curves are displayed for continuous evaluation with 6-hour horizon ("Continuous"), peak score ("Peak score"), and fixed horizon ("Fixed horizon"). For each strategy, the solid line shows the net benefit of triggering an alert when the predicted sepsis risk exceeds a given threshold probability, where net benefit represents the proportion of true-positive alerts minus a threshold-dependent penalty for false-positive alerts. Dashed lines show the corresponding "Treat All" baselines for each strategy, and the dotted grey line indicates the "Treat None" strategy (net benefit = 0). Note that Peak Score and Fixed Horizon share the same "Treat All" baseline as the prevalence is the same in both strategies. Shaded areas denote 95% confidence intervals.*

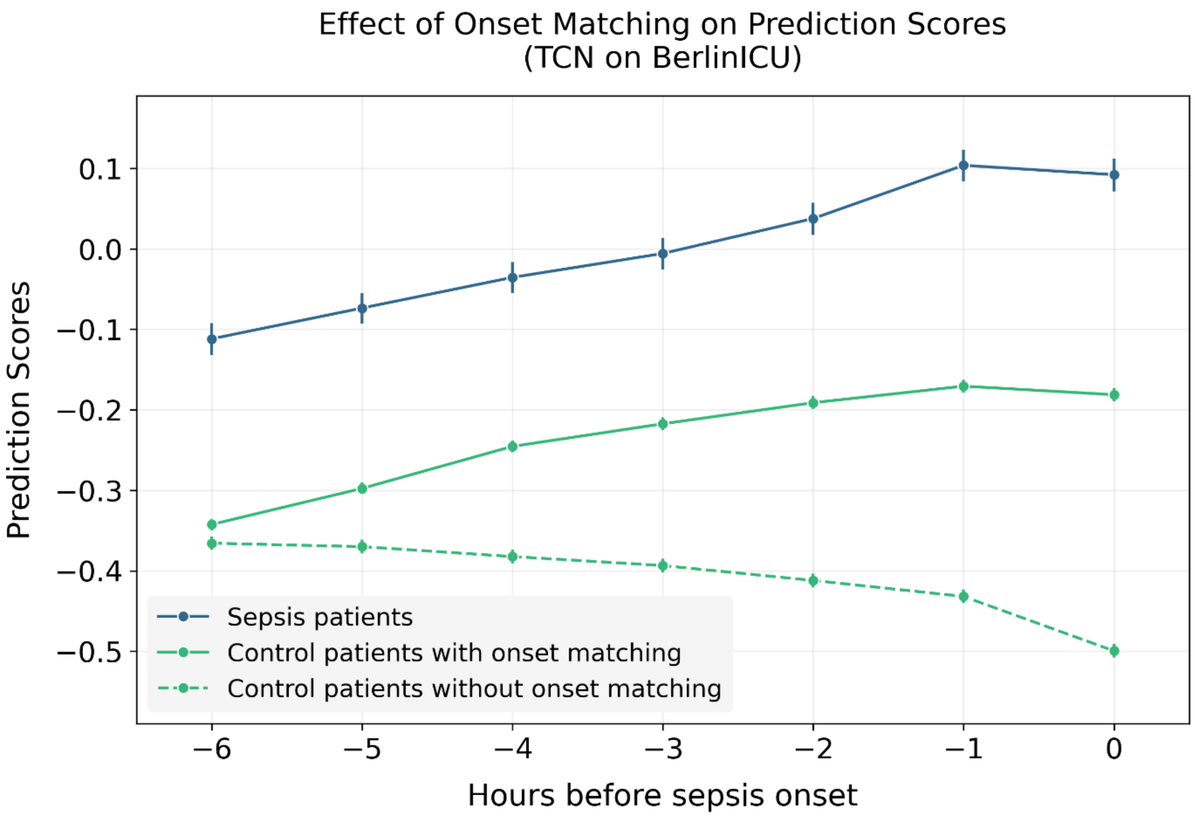

Figure S10: Temporal Convolutional Network (TCN) model output over time for sepsis patients, matched controls, and unmatched controls*. Time is anchored at sepsis onset (cases) or at the last available data point, either at discharge or at 7 days after admission, whichever occurred first (controls). The plot shows the output of the TCN models for cases, and matched and unmatched controls. For sepsis patients, predicted scores remain elevated up to onset. For controls, unmatched patients exhibit a clear reduction in predicted risk toward discharge, while matched controls remain at higher average risk levels, closer to those of sepsis patients. This effect highlights how inclusion of discharge-proximal time points for unmatched controls inflates performance when onset matching is not applied. Error bars: 95% confidence interval.*
